# Individual differences in word skipping during reading in English as L2

**DOI:** 10.3758/s13423-024-02529-w

**Published:** 2024-06-12

**Authors:** Diana Esteve, Manuel Perea, Bernhard Angele, Victor Kuperman, Denis Drieghe

**Affiliations:** 1 Universitat de València, Valencia, Spain; 2 Universidad Nebrija, Madrid, Spain; 3 Bournemouth University, Poole, UK; 4 McMaster University, Hamilton, Canada; 5 University of Southampton, Southampton, UK; 6 University of Cambridge, Cambridge, UK

**Keywords:** Eye movevements, Reading, Word skipping, Bilingualism

## Abstract

The Multilingual Eye-movement Corpus (MECO; Siegelman et al., 2022) contains data from unbalanced bilinguals reading in their first language (L1) for a variety of languages and in English as their second language (L2). We analyzed word skipping in L2 on the basis of five predictors consisting of the frequency and length of the word in L2 and three measures of individual differences. Besides the L2 proficiency of the participant, two novel measures were also constructed: the average amount of skipping in L1 across participants per language and whether an individual reader skips words often in their L1 compared with other L1 readers in the same language. Word skipping in L2 increased for short and high-frequency words, for participants with higher L2 proficiency, for readers whose L1 featured relatively high average skipping rates compared with the other languages, and especially for participants who skip more often in L1 than their peers. All three individual differences interacted with word length such that their influence was more pronounced for longer words. Our results show that readers prefer to maintain a certain level of word skipping resembling how they read in L1. Due to lower L2 than L1 proficiency in unbalanced bilinguals, word skipping in L2 would often be based on a comparatively less advanced stage in parafoveal word recognition.

## Introduction

Readers sample information from a line of text through a series of fixations, during which the eyes are comparatively still and information is extracted, and saccades, which are fast, jerk-like movements that bring new information into the centre of the visual field where acuity is highest (the fovea). These eye movements are tightly linked to the cognitive processing of the reading material (Liversedge & Findlay, 2000). Indeed, numerous eye-tracking studies indicate that words that are easier to process will be fixated upon for a shorter time than difficult words (for a review, see Rayner, 2009). Fixation times on words that are either short, high-frequency, or predictable from the preceding context are, therefore, typically shorter than on long, low-frequency, or unpredictable words.

A striking observation in reading is that not all words are directly fixated. A word is *skipped* when the word does not receive a direct fixation during the first pass (first-time reading). Skipping is common; approximately one in every three words is skipped (Rayner, 1998). Similar to fixation times, words that are easier to process, such as short, high-frequency, and predictable words, are skipped more often (e.g., Brysbaert et al., 2005) than their more difficult counterparts. It is, therefore, tempting to assume that manipulations that impact fixation times will have a similar influence on word skipping. However, several studies observed differential impacts of various manipulations on word skipping and fixation times (e.g., Drieghe, 2008), raising questions about whether these phenomena reflect similar processing of the material being read.

A critical question in reading research is what degree of processing has been achieved when a reader decides to skip a word. In serial models of eye movements during reading, such as the E-Z Reader model (Reichle et al., 1998), only one word is lexically processed at a time. When a word *n* has been lexically identified, attention shifts to the next word. The arrival of the attentional beam on word *n* + 1 usually precedes the arrival of the eyes, and it is during this time window that parafoveal processing occurs. E-Z Reader states that if an advanced stage of the processing of the parafoveal word is reached early enough, a signal is sent to replace the programming of a saccade towards word *n* + 1 with a saccade towards word *n* + 2. In short, E-Z Reader assumes a word is skipped because an advanced stage in the word recognition of word *n* + 1 is reached, and this will happen more often for words that are easy and therefore faster to process. Likewise, models that do not impose serial lexical processing, such as the SWIFT model (Engbert et al., 2005), often also assume that the majority of word *n* + 1 processing has finished when the saccade targeting system decides to skip it.

A somewhat alternative approach is offered by the Extended Optimal Viewing Position (EOVP) model (Brysbaert & Vitu, 1998), which assumes that skipping mostly reflects an educated guess. This guess would be primarily based on parafoveal word length and an estimation that the word that will be skipped will often be either recognized by the time the eyes land on word *n* + 2 or that skipping word *n* + 1 will not hinder overall text understanding. For example, a three-letter word may be skipped not because it is recognized whilst fixating on the previous word but because experience has taught the reader that they will recognize a three-letter word before landing on the word following it or that not identifying it will not result in a high cost for comprehension. The latter scenario would rely less on advanced parafoveal processing than assumed by, for instance, the E-Z Reader model. Importantly for the current study, the EOVP model suggests a “habit” when reading that predicts skipping rates of words of a certain length.

Despite the complexity of various reading models and their ability to simulate many eye movement behaviours, many still present significant limitations. Currently, E-Z Reader and most other reading models focus solely on monolingual reading, despite 74% of adults in the European Union knowing more than one language (Eurostat, 2024). Moreover, models usually ignore individual differences in reading, such as differences in reading ability (Everatt et al., 1998). The current study, therefore, investigates individual differences in a bilingual context by examining word skipping in a second language (L2) and asks the novel question of whether individual differences, including eye movement behaviour in L1, can predict the skipping of a word in L2.

Individual differences are very pronounced in word skipping during reading, with some participants skipping almost every other word and others hardly ever skipping. We were interested in three specific individual differences. The first is the L2 proficiency of the reader. In the Ghent Eye-tracking Corpus (GECO), in which unbalanced bilinguals read half a novel in Dutch (L1) and the other half in English (L2), Cop et al. (2017) observed lower skipping rates for the same participants in their L2 compared with in their L1. A possible explanation could be L2 proficiency. Higher proficiency in L2 is associated with faster lexical processing and, therefore, a skipping behaviour that resembles L1 skipping more.

Before introducing the other two measures of individual differences we investigate, we describe the database we will use. To examine differences between L1 and L2 skipping across the same participants, we use the Multilingual Eye-movement Corpus (MECO; Siegelman et al., 2022). The MECO includes data from 13 different L1 (Dutch, English, Estonian, Finnish, German, Greek, Hebrew, Italian, Korean, Norwegian, Russian, Spanish, and Turkish). Furthermore, the MECO includes data for (most) participants reading in English as L2 (Kuperman et al., 2022), thereby directly comparing L1 and L2 reading for the same participants. Siegelman et al. (2022) found substantial differences in average rates of word skipping between languages in L1 but also determined that the effect was explained by differences in average word length between languages. Thus, a language with longer words, on average, will feature less word skipping than a language with comparatively shorter words.

Returning to skipping in L2, we were intrigued by the assumption of the EOVP model that skipping would involve an educated guess based on parafoveal word length. If this were the case, would the specific parameters of an individual for this estimated guess be similar in L1 and L2? Therefore, the second individual difference we will examine is the average skipping rate across subjects per language. Do participants who are used to often skipping in their L1 (due to an abundance of relatively shorter words) show a similar tendency to skip words frequently in English (their L2) compared with participants whose L1 features, on average, longer words? Finally, the third individual difference we report is whether an individual who is a frequent skipper in their L1, compared with their L1 peers, is also likely to be a frequent skipper in their L2.

The suggestion of skipping rates being influenced by factors other than processing speed is also compatible with intriguing findings reported by Rayner et al. (2006) on skipping rates in older versus younger adults. They observed that older adults skipped words more often than younger readers, even though the speed of lexical processing is known to slow down with age. Rayner et al. proposed that older adults adopted a “risky” reading strategy, with more skips and more regressions within the text. Older adults would compensate for slower processing by using lexical and contextual knowledge to try to guess the words they are skipping. Whereas this risky reading strategy is not universally accepted (McGowan & Reichle, 2018) and might be restricted to alphabetical languages (Zhang et al., 2022), it could be tied to the “educated guess” assumption (Brysbaert et al., 2005), with older readers having more experience guessing when a word can be safely skipped. Likewise, L2 readers might employ similar strategies to maintain a skipping rate somewhat close to what they are used to in L1, even though word processing in L2 would also be comparatively slower.

Our research question is therefore, to what degree do the individual differences defined above influence the probability of word skipping in L2 (English in this case)? Besides these factors, we include word length and frequency in L2 as predictors, given their well-established influence on skipping rates.^1^ We predict higher skipping rates in L2 for shorter and more frequent words. We also predict higher rates of L2 skipping for participants with a high L2 proficiency, as well as those whose L1 features higher average skipping rates compared with other L1s and those who, compared with their L1 peers, skip relatively often in L1.

## Method

### Dataset

Participants in the MECO read short, Wikipedia-style texts both in their L1 and L2 whilst their eye movements were recorded. Only the MECO data for participants who completed both L1 and L2 studies were used for this study. This resulted in 11 languages in the analysis (Greek, Spanish, Hebrew, Turkish, Estonian, Finnish, Norwegian, Italian, German, Dutch, and Russian) out of the 13 languages present in the MECO, as the South Korean sample did not participate in the L2 part of the study, and the English sample had English as their L1. A more detailed description of the MECO corpus, its construction, and its structure can be found in Siegelman et al. (2022) for L1 reading and Kuperman et al. (2022) for L2 reading data.

Data trimming followed the procedures described by Kuperman et al. (2022) and included eliminating observations with very short (<80 ms) or very long (above 99th percentile for the participant) fixation times. Words above the 90% percentile in length were additionally eliminated to prevent a very small number of unusually long words with only a few occurrences in the corpus from influencing the results excessively. This resulted in only words up to eight letters included in the analysis. Participants who did not complete all three relevant L2 proficiency measures (detailed below) were also excluded. Our final dataset contained 402 participants and 440,824 observations from the initial database of 543 participants with L2 data and 669,364 observations. We obtained the word length and frequencies of the L2 words in the MECO corpus from Kuperman and Siegelman (2023).

There is no clear consensus in reading research on how to operationalize L2 proficiency. Measures previously used included multiple-choice comprehension questions (Huang & Jiang, 2022), only one type of measure such as lexical knowledge or vocabulary (Dirix et al., 2020; Rets & Rogaten, 2021), a combination of measures from the same test battery (Berzak et al., 2022) or a combination of several different tests (Kuperman et al., 2022; Nisbet et al., 2021; Parshina et al., 2022). The current study did not aim to analyze differences between specific L2 proficiency measures (e.g., vocabulary, grammar) but to examine general L2 proficiency related to word skipping. To that end, a composite measure of L2 proficiency was created by selecting relevant measures in the MECO (Kuperman et al., 2022): spelling, vocabulary, and lexical knowledge. S*pelling* was assessed by the Spelling Recognition Test (adapted from Andrews & Hersch, 2010); *vocabulary*, from the Vocabulary Knowledge Test (adapted from Nation & Beglar, 2007); and *lexical knowledge* from the answers to the Lexical Test for Advanced Learners of English (LexTALE; Lemhöfer & Boersma, 2012). The scores were converted into a percentage of correct answers per test—similar to Berzak et al. (2022)—and averaged. This procedure also averted multicollinearity issues due to medium to high correlations in the test scores that make up the composite measure (see Table 1).

**Table 1:** Pearson correlations and descriptive statistics for L2 proficiency measures across all L2 subjects (*N* = 543)

Variable	*n*	*M*	*SD*	1	2
1. Spelling	539	79.51	12.08	_	_
2. Vocabulary	536	75.46	24.66	.40***	_
3. Lexical knowledge	537	76.28	12.05	.58***	.63***

^*^p < .05. **p < .01. ***p < .001.

Whether participants skipped often in their L1 compared with their L1 peers was operationalized by calculating the mean individual skipping rate in L1 centered on the mean skipping rate and scaled on the standard deviation of all participants in that L1. Finally, the mean skipping rate per language in L1 was represented by the mean skipping rate per L1 for all subjects in that language, centered on the mean skipping rate in English as L1. In other words, a high value meant participants from this specific L1 skipped on average more often than the L1 readers in English.

### Analyses

A generalized linear mixed model (GLMM) was chosen to predict L2 skipping rates. Data were analyzed with the lme4 (Version 1.1-29) package (Bates et al., 2015) for R (Version 4.2.2). We did not have strong predictions for many of the interactions, so we adopted an exploratory model-building strategy for finding the best-fitting model. Table 2 lists the fixed structure of the initial model. The initial model has intercepts only for subjects and items as random factors. Next, interactions were introduced to the fixed structure of the model. In every step, we added a two-way interaction. If the model converged with the additional interaction whilst improving the fit of the model, it remained in the model. We first added interactions with L2 proficiency, starting with the fixed factor with the highest *z*-value, then the second highest, and so on. After L2 proficiency, interactions amongst the other fixed factors were added. No models with a three-way interaction (or higher) converged. After nothing could be added to the fixed structure, we tried adding slopes to the random structure to obtain the maximum structure our data allowed. The only model that converged and resulted in a better fit compared with the intercept-only model included an additional word length slope for the subjects’ random effect.

**Table 2:** Predictor in the model

Variable name	Description
L1 skipping	Mean individual skipping rate in L1 centered on the mean skipping rate and scaled on the *SD* of all participants in that L1 (e.g., if the participant has an L1 skipping value of 0.1, they skip 10% of an *SD* more than other participants in their L1).
Mean skipping per language	Mean skipping rate per L1 for all subjects, centered on mean skipping rate in English (e.g., if a language has 0.1 mean skipping rate, speakers skip 10% more on average than English speakers).
Length	Number of letters in the specific word in the L2 texts, centered (e.g., a value of 3 means the word has three letters more than the average, which is 4.32 letters).
L2 proficiency	Proficiency of a participant in English as their L2, scaled.
Frequency	Zipf frequency of the word in L2, centered (e.g., van Heuven et al., 2014)

## Results

**Fig. 1:**
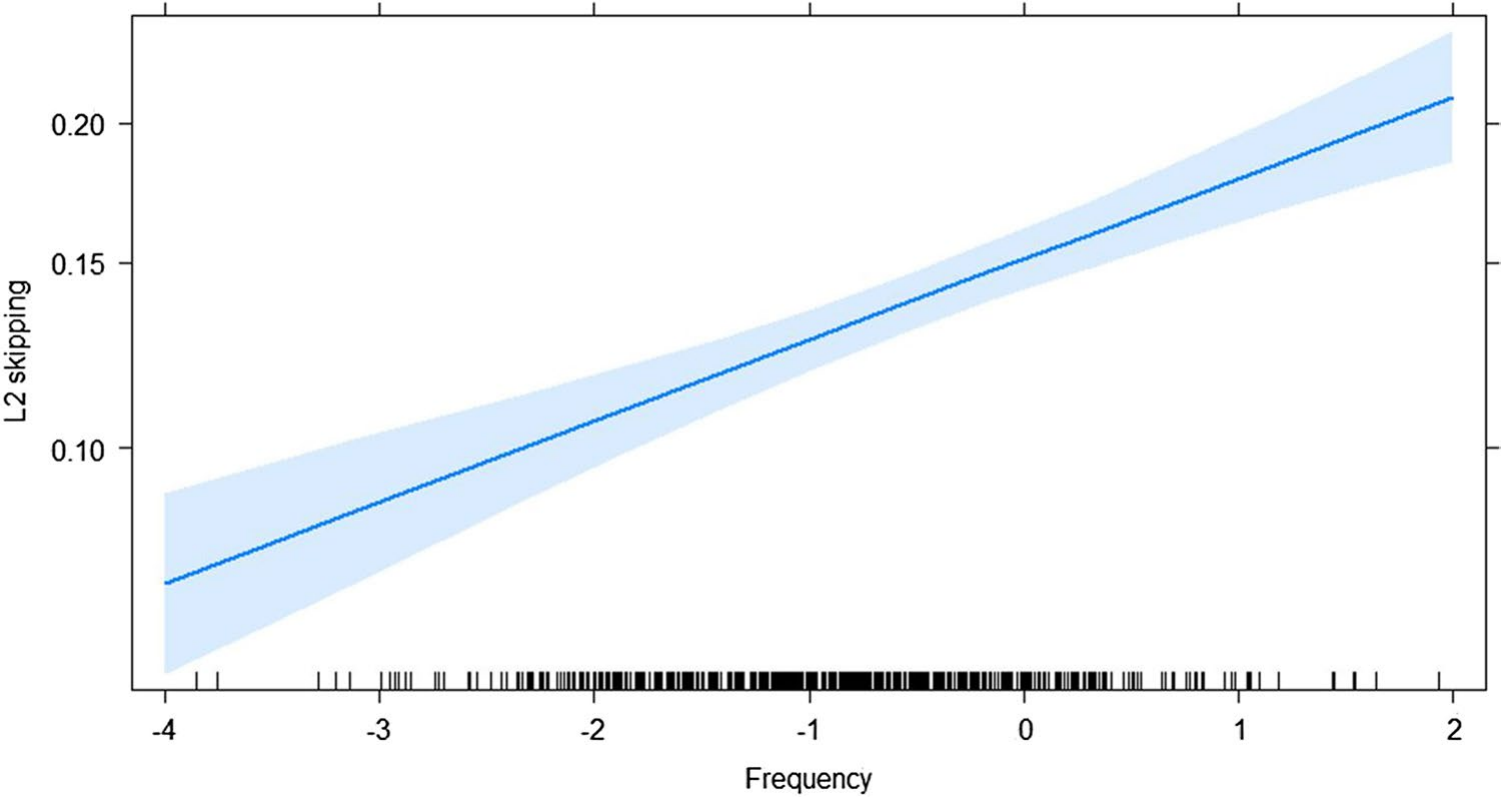
Predicted L2 skipping rate as a function of L2 word frequency (centered). The grey band is the 95% confidence interval. Individual observations are plotted as black ticks on the *x*-axis

**Fig. 2:**
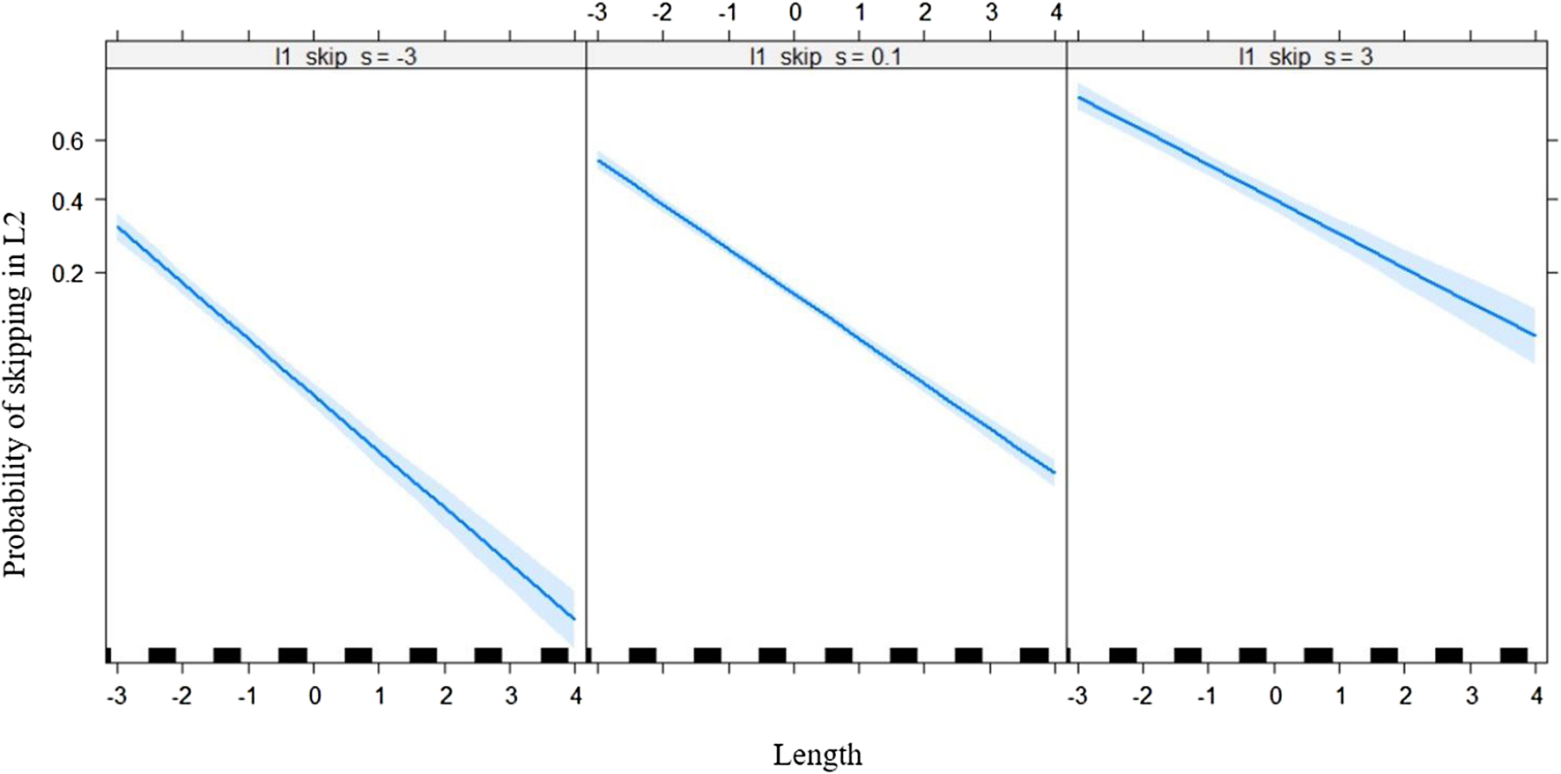
Predicted L2 skipping rate as a function of word length (centered) and skipping in L1 (centered). The left panel corresponds to participants who are less frequent skippers in L1 compared with their L1 peers, and the right panel corresponds to participants who are frequent skippers in their L1. The grey band is the 95% confidence interval. Individual observations are plotted as black ticks on the *x*-axis centered on the mean word length (4.32 letters)

**Fig. 3:**
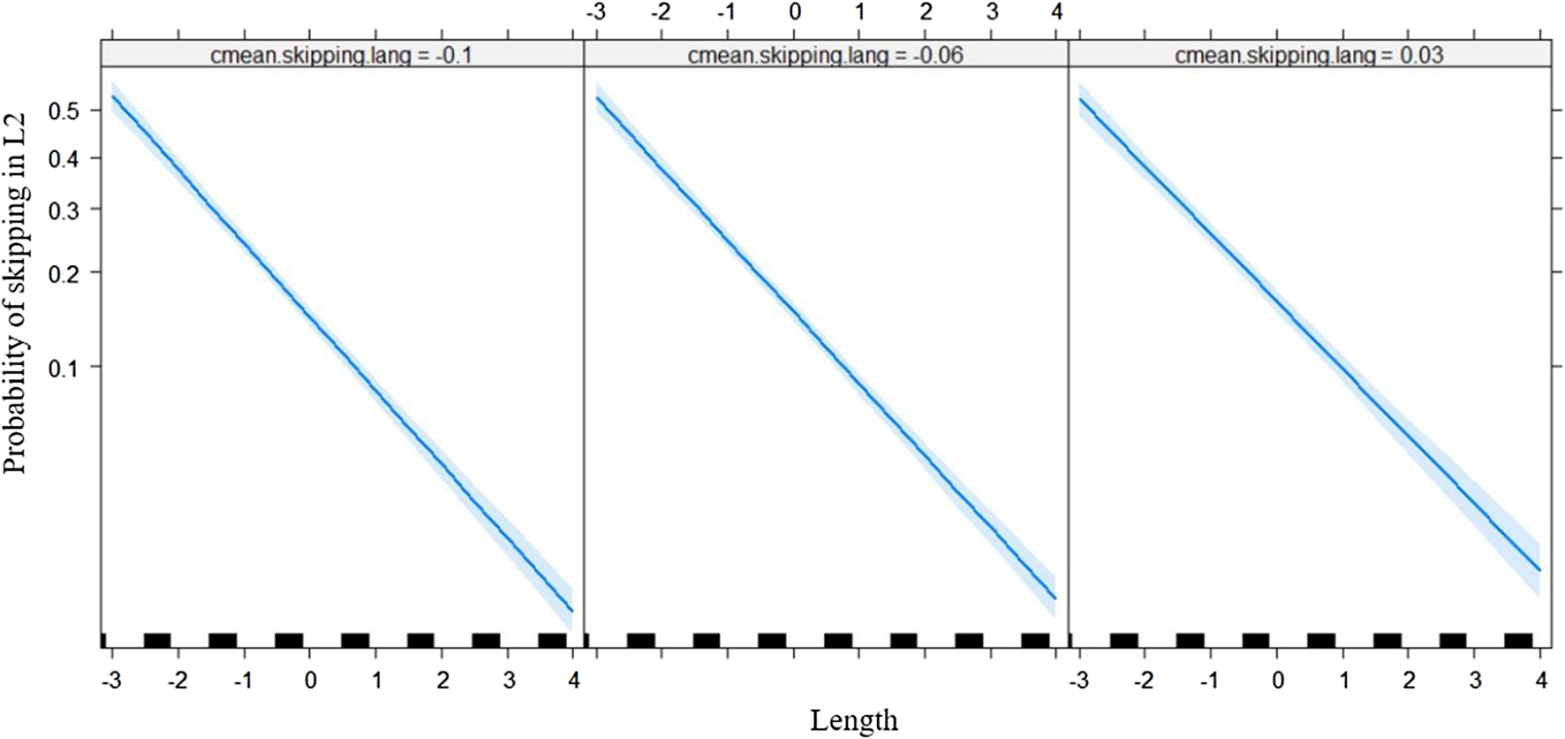
Predicted L2 skipping rate as a function of word length (centered) and skipping in L1 (centered). The left panel corresponds to participants from an L1 who, on average, skip less often compared with participants who have English as L1, and the right panel corresponds to participants from an L1 where average skipping is higher compared with English. The grey band is the 95% confidence interval. Individual observations are plotted as black ticks on the *x*-axis centered on the mean word length (4.32 letters)

**Fig. 4:**
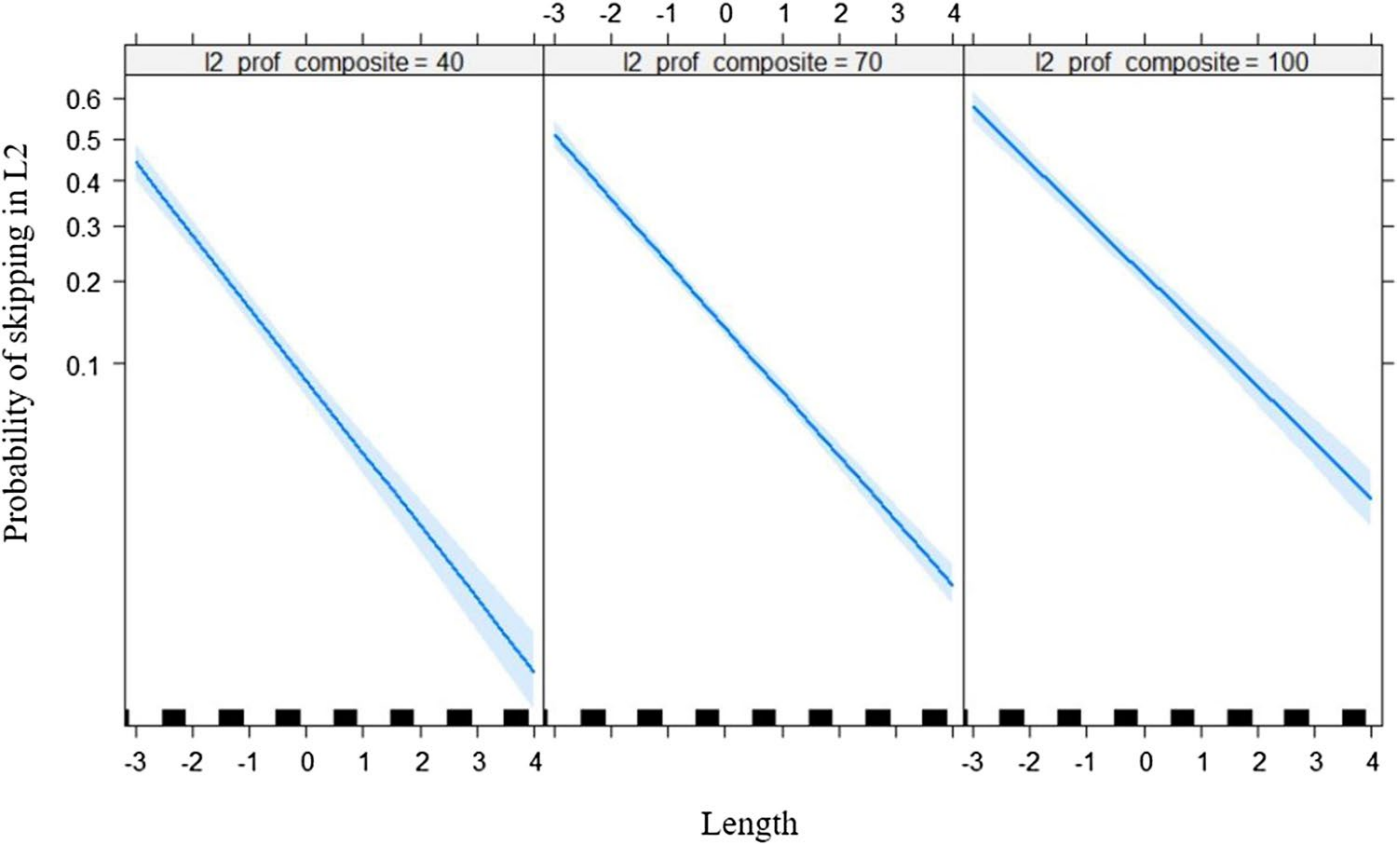
Predicted L2 skipping rate as a function of word length (centered) and L2 proficiency (centered). The left panel corresponds to participants who are less proficient in L2, and the right panel corresponds to participants who are more proficient in L2. The grey band is the 95% confidence interval. Individual observations are plotted as black ticks on the *x*-axis centered on the mean word length (4.32 letters)

Table 3 shows the output of the LMMs. A main effect of frequency was observed such that high-frequency words were skipped more often than low-frequency words (Fig. 1). Word length had the expected main effect whereby short words are skipped more often but was qualified by three two-way interactions. The interaction of word length with L1 skipping (Fig. 2) showed that readers who were comparatively frequent skippers in L1 were also typically frequent skippers in L2, especially for long words. The mean skipping per language had a small but significant effect in that readers from an L1 where skipping was comparatively more frequent also skipped more often in L2. However, this effect was qualified by an interaction with word length (Fig. 3), such that this was only true for longer words. Finally, effects of L2 proficiency were observed in the expected direction of highly proficient L2 readers skipping more often. Again, this effect was qualified by word length (Fig. 4) in that the effect of L2 proficiency was more pronounced for longer words.

**Table 3:** Fixed effects structure of the logistic linear mixed model

	Estimate	*SE*	*z* value
(Intercept)	−1.666	0.042	−39.359
L1 skipping	0.443	0.024	18.548
Length	−0.588	0.022	−27.052
Mean skipping per language	1.039	0.331	3.113
L2 proficiency	0.218	0.024	9.179
Frequency	0.202	0.031	6.562
Length × L1 skipping	0.049	0.010	4.906
Length × Mean Skipping per Language	0.405	0.159	2.546
Length × L2 proficiency	0.034	0.010	3.459

All |z| > 1.96 are statistically significant.

## Discussion

**Fig. 5:**
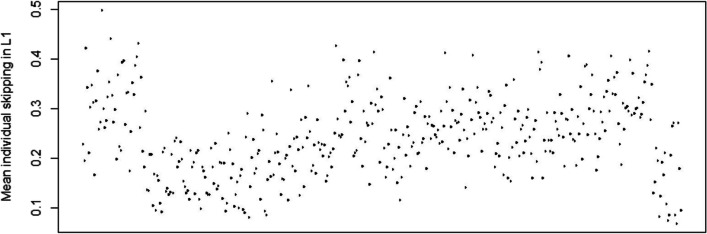
Mean skipping rate in reading in L1 for all participants across all languages. The ordering on the *x*-axis is arbitrary (based on participant file name)

Few individual differences are as pronounced during reading as word skipping. Some readers hardly skip any words, whereas others skip almost every other word (Fig. 5). In this study, we examined word skipping behaviour of participants reading in English as their L2. We were particularly interested in whether we could predict L2 skipping from three measures of individual differences. Firstly, a composite measure for L2 proficiency was constructed based on a selection of tests that are part of the MECO corpus (Kuperman et al., 2022). The MECO also allowed us to directly compare the eye movement behaviour of participants reading both in their respective L1 and in English as L2. Two novel measures were constructed: (1) how often participants, on average, skip in their L1 compared with English L1 readers and (2) properties of the individual participant, being how often a participant skips in L1 compared with their peers. Our results were very straightforward. Besides the well-documented effects of word length and frequency whereby short and high-frequency words are skipped more often than long and low-frequency words, we observed significant effects for all our individual measures: Word skipping increased for participants with higher L2 proficiency, for participants whose L1 featured higher skipping rates on average compared with the other languages, and for participants who, in their L1, skip more often compared with their peers. Before we go into these three effects in detail, we should mention that they were all qualified by a similar two-way interaction with word length such that their impact was more pronounced for longer words. This is important as it suggests these individual differences matter more for words that are difficult to process than for easy words.

Most of the models of eye movements during reading discussed in the Introduction assume that when the saccade targeting system decides to skip a word, a fairly advanced stage in the word recognition of that word has been reached. Cop et al. (2017) observed lower skipping rates in L2 compared with L1, which could be a consequence of the participants in their study being unbalanced bilinguals with a lower L2 proficiency than L1 proficiency. Although the models mentioned were not designed to account for bilingual readers, the influence of L2 proficiency can be considered compatible with most of them if we assume that lower L2 proficiency would result in slower word processing and, therefore, reduced word skipping. The observation that the impact of L2 proficiency was more pronounced for longer and, therefore, more difficult words is compatible with studies showing similar effects for word frequency. In the literature, the frequency effect in reading in L1 tends to be smaller for more proficient readers; specifically, less skilled readers show proportionally greater costs on the reading times of low-frequency words (e.g., Ashby et al., 2005). In other words, less skilled readers are proportionally more slowed down by difficult words than are better readers. Similarly, the reduced skipping for longer words was more pronounced for readers with comparatively lower L2 proficiency. Note, however, that, unlike the L1 proficiency in the previous studies mentioned above, L2 proficiency did not interact with word frequency in our study. This fits with the results of an analysis of the L2 frequency effect on reading times in the GECO corpus (Cop et al., 2015), which included both L1 (Dutch) and L2 (English) proficiency measures. It showed that L1 proficiency was a significant predictor of the frequency effect in L2, but L2 proficiency was not (but see Mor & Prior, 2022, for findings with Hebrew–English bilinguals). As the MECO does not include measures of L1 proficiency, further research will be needed to see if the findings from Cop et al. generalize to word skipping. To interpret the interaction between word length and L2 proficiency, we mostly focus on the notion that less proficient readers are proportionally more slowed down by difficult words, which in our experiment would be the longer words.

The effects of the average skipping rate per language and individual skipping profile in L1 on skipping in L2 are related in that both reflect a “habit” carrying over from L1 into L2. The effect of the average skipping rate in L1 is that readers who are used to skipping more frequently on average^2^ will skip slightly more often in L2. One could link these findings to the observation of a preferred saccade length (PSL), which is the distance between the saccade launch site and saccade target when participants neither undershoot nor overshoot the target word (McConkie et al., 1988). In English, the PSL is estimated to be around seven characters. If the PSL is a fixed value as assumed by, for instance, E-Z Reader (Reichle et al., 1998), which uses this parameter to estimate the likelihood of under- and overshooting a saccade target, some spillover into eye movement behaviour in L2 is plausible. However, research by Cutter et al. (2018) indicates that this parameter can be adjusted on a trial-by-trial basis when participants are confronted with materials that vary in word length properties and, therefore, that the PSL is not fixed. Moreover, it is important to point out that the effect of the average skipping rate in L1 is numerically minimal, and the interaction is such that it only applies to long words (see Fig. 3). A more parsimonious interpretation would be that readers not used to encountering particularly long words in L1 will be especially hesitant to skip them in L2 (even though they could adjust saccade length if they wanted to, as per Cutter et al., 2018).

Whereas the effect of the average skipping rate in L1 per language on L2 skipping is modest and probably can only be detected with the statistical power of a corpus study such as the MECO, the effect of how often a participant skips in L1 compared with their peers is quite substantial. The effect size is also compatible with the surprisingly high within-participant correlation observed between L1 and L2 skipping rates in the MECO of 0.69 (Kuperman et al., 2022). As shown in Fig. 2, a participant’s relative skipping rate in L1 compared with their peers carries over into L2, and our model indicates that this is not modulated by L2 proficiency. This suggests that readers adopt a “habit” of how often they usually skip and try to maintain this habit to a certain extent even when reading is more effortful, such as when L2 proficiency is lower than L1 proficiency in unbalanced bilinguals. This is similar to Rayner et al.’s (2006) finding that older readers do not reduce their skipping rate compared with younger readers (potentially, they even increase it) even though word processing is slowed down due to age (Rayner et al., 2006).

Whereas effects of L2 proficiency can be considered compatible with most models of eye movements, the influences of L1 average skipping per language and the L1 skipping profile of an individual probably cannot. Even though word skipping is often assumed to be contingent on reaching an advanced stage in the word recognition of the target word (Drieghe et al., 2005), we also observed a tendency to maintain the same familiar skipping rate despite suboptimal conditions (e.g., L2 word processing in unbalanced bilinguals). Maintaining a habit of skipping words of a certain length even when word processing speed is reduced in L2 appears more compatible with a model that assumes skipping is based on an educated guess, such as the EOVP model (Brysbaert, et al., 2005). However, it is important to note that simulations would need to establish whether models can simulate the observed patterns and that the EOVP model is not computationally implemented.

In conclusion, L2 skipping is influenced not just by L2 proficiency but also by how often the individual skips in L1 (compared with other L1 readers) and, to a lesser extent, by the average L1 skipping rates per language. Whereas bilingual models do exist for isolated word recognition (Bilingual Interactive Activation plus model; van Heuven & Dijkstra, 2010), our research highlights the need for multilingual reading models. It also shows how research into individual differences in reading can provide new insights into well-studied phenomena. Future research will need to examine how individual differences in other effects, such as predictability, carry over from L1 to L2. Finally, our findings challenge the idea that skipping is always based on parafoveal processing. Instead, readers prefer to maintain a certain level of word skipping even when word processing is comparatively slowed down.

## Data Availability

The raw data files used for the statistical analyses are publicly available online (https://osf.io/uv8w2/). This study was not preregistered.

## Footnotes

1 As there are no published sentence completion rates for the texts that make up the MECO corpus, predictability was not entered into the model. While future research should address this limitation, it is important to note that in a corpus of natural language, predictable items tend to be rare (Luke & Christianson, 2016) and that therefore—although not ideal—analyses of corpus studies often do not feature a measure of predictability (e.g., the GECO corpus; Cop et al., 2017). Conditional probability distributions based on language models might provide a more pragmatic alternative for the labour-intensive collection of sentence completion ratio’s and are currently available for the MECO materials read in L1 (de Varda & Marelli, 2023) but not yet for the materials read in English as L2 (note that the materials read in English as L1 were different from the materials read in English as L2 in the MECO).

2 As expected, our measure of the skipping rate for all participants in L1 compared with English as L1 was strongly correlated with average word length in L1 (−.77). This relationship was expected based on previously reported findings in the MECO that average skipping rates in L1 reflect word length properties of that L1 (Siegelman et al., 2022).

## Code availability

The R scripts used for the statistical analyses are publicly available online (https://osf.io/uv8w2/). This study was not preregistered.
